# Effective Colorectal Cancer Screening—Adherence is Key

**DOI:** 10.1002/ueg2.70180

**Published:** 2026-01-24

**Authors:** Hermann Brenner, Michael Hoffmeister

**Affiliations:** ^1^ Cancer Prevention Graduate School German Cancer Research Center (DKFZ) Heidelberg Germany; ^2^ Division of Clinical Epidemiology of Early Cancer Detection German Cancer Research Center (DKFZ) Heidelberg Germany

Colorectal cancer (CRC) is the second most common cancer related cause of death globally, accounting for close to one million deaths each year [1]. The majority of these deaths could be prevented by effective screening. Best established and most widely recommended screening strategies include colonoscopy every 10 years, flexible sigmoidoscopy every 5 years, and annual or biennial fecal immunochemical tests (FITs). Screening programs offering one or several of these screening tests have been implemented or are currently being implemented in an increasing number of countries [2].

Screening colonoscopy is the approach most reliably detecting CRC and its precursors, and immediate removal of these precursors yields strong preventive effects, besides early detection of already prevalent CRC [3]. However, screening colonoscopy requires comprehensive bowel cleansing and major health care capacities and resources which are lacking in many countries, even within Europe. Regular screening by FIT and referring to colonoscopy only the minority of people with a positive FIT result therefore seems to be a prudent approach to use limited capacities more efficiently. Another common expectation is that the less invasive screening by FIT would be taken up by a larger share of the eligible population, thereby compensating for the lower sensitivity to detect CRC and its precursors compared to colonoscopy and achieving similar preventive effects on the population level.

It has been unclear, however, to what extent this expectation is met in practice, that is, if FIT‐based screening would indeed not perform significantly worse with respect to reducing CRC risk and mortality than screening by colonoscopy, despite the lower sensitivity of FIT. This question has been addressed, for the first time, in a randomized trial design in the COLONPREV trial in Spain [4, 5], and further trials comparing performance of colonoscopy‐ and FIT‐based screening are under way in the US and Sweden [6, 7]. The COLONPREV trial demonstrated that, with the screening use achieved in this trial (FIT group: 39.4% FIT and 0.5% colonoscopy use; colonoscopy group: 20.1% colonoscopy and 11.7% FIT use), the FIT‐based screening offer was non‐inferior to the colonoscopy‐based screening offer with respect to reduction of CRC‐related mortality. Subgroup analyses presented in the current issue of the journal confirmed non‐inferiority across sex and age subgroups.

It should be noted, that these results should be considered preliminary, as longer follow‐up than 10 years is needed to disclose the full impact of CRC screening programs. Furthermore, generalization of the results needs to be done with caution as many countries now use much higher FIT cutoffs (up to 120 μg/g) than those used in the trial (15 μg/g for initial and 20 μg/g for subsequent FITs). Most importantly, however, effectiveness of either type of screening offer, and hence also inferiority or non‐inferiority of one type compared to the other, strongly depends on the achieved adherence rates, which may vary widely depending on specific features of the screening offer. Of note, much higher adherence to FIT‐based screening (well above 60%) than observed in the COLONPREV trial has meanwhile been achieved in a number of well‐organized national screening programs, such as those in the Netherlands, Denmark, Scotland or Sweden [8]. A key implementation feature of these programs is that FITs are directly sent, along with well‐designed invitation and information materials to the target population, whereas use of either screening modality in the COLONPREV trial required an appointment to a local screening office. For screening colonoscopy, also much higher adherence than observed in the COLONPREV trial might be achieved in well‐organized settings. This has been demonstrated in the Norwegian arm of the NordICC trial, in which adherence to the screening colonoscopy offer was 60.7% [9]. At the same time, the NordICC trial also demonstrated major variability in screening colonoscopy adherence according to country‐specific or organizational circumstances, as reflected in the strong variation of adherence across the four participating countries (Norway: 60.7%, Sweden: 39.8%, Poland 33.0%, Netherlands 22.9%, *p* < 0.001).

In conclusion, while there are several evidence‐based options for effective CRC screening, the key for their impact on the population level is high adherence to high‐quality screening offers. Such implementation is of paramount importance for preventing the increasing number of CRC cases that otherwise is to be expected in the decades to come due to demographic developments [10]. The COLONPREV trial has demonstrated that a FIT‐based screening program was not inferior to a colonoscopy‐based screening program across sexes and age groups. Further efforts should focus on ways to optimize implementation of highly effective and cost‐effective screening programs that achieve substantially higher adherence rates than those observed in the COLONPREV trial.

## Conflicts of Interest

The authors declare no conflicts of interest.

## Data Availability

The authors have nothing to report.
